# Processing Ultrasound Scans of the Inferior Vena Cava: Techniques and Applications

**DOI:** 10.3390/bioengineering10091076

**Published:** 2023-09-12

**Authors:** Piero Policastro, Luca Mesin

**Affiliations:** Mathematical Biology and Physiology, Department Electronics and Telecommunications, Politecnico di Torino, 10129 Turin, Italy; piero.policastro@polito.it

**Keywords:** ultrasound, cardiology, inferior vena cava, caval index, veins, blood vessels, active contour, deep learning, POCUS

## Abstract

The inferior vena cava (IVC) is the largest vein in the body. It returns deoxygenated blood to the heart from the tissues placed under the diaphragm. The size and dynamics of the IVC depend on the blood volume and right atrial pressure, which are important indicators of a patient’s hydration and reflect possible pathological conditions. Ultrasound (US) assessment of the IVC is a promising technique for evaluating these conditions, because it is fast, non-invasive, inexpensive, and without side effects. However, the standard M-mode approach for measuring IVC diameter is prone to errors due to the vein movements during respiration. B-mode US produces two-dimensional images that better capture the IVC shape and size. In this review, we discuss the pros and cons of current IVC segmentation techniques for B-mode longitudinal and transverse views. We also explored several scenarios where automated IVC segmentation could improve medical diagnosis and prognosis.

## 1. Introduction

The cardiovascular system is essential for the delivery of nutrients and the removal of waste products from the body, as well as for the functioning of the immune system. Its importance is underlined by the fact that cardiovascular diseases cause approximately 31% of deaths worldwide, and the majority of hospitalised patients require fluid therapy [1,2].

Arteries carry blood from the heart to the tissues and are subject to high blood pressure. Typically, the geometry of these vessels is regular. An altered shape or partial occlusion is an indicator of health problems [3,4].

Veins, on the other hand, are vessels that return to the heart. In the systemic circulation, they collect deoxygenated blood and waste products from the tissues and carry them to the heart. Compared to arteries, veins have a thinner tunica media (the middle layer of the vessel wall). Due to their structure, the shape of the veins is more prone to change over time and is mainly influenced by external pressure (in fact, the dynamics of the blood vessel walls is controlled by the transmural pressure, defined as the difference between the internal and the external pressures) [5]. Veins are blood reservoirs and contain approximately 70% of its total volume [6,7]. Assessment of blood volume status, which is primarily reflected in the veins, is required to properly plan the treatment of patients admitted and managed in the cardiology, emergency, and internal medicine departments, as well as in general practice [8].

The biggest vein in our body is the inferior vena cava (IVC). It is positioned in the abdominal cavity; then, it passes through the thoracic diaphragm at the caval opening and connects to the right atrium. The right atrial pressure (RAP), fluid distribution in the venous compartment, and hydration status of the patient are all related to IVC size and collapsibility [9,10].

Assessment of the IVC using ultrasound (US) scans is a promising technique because it is fast, non-invasive, cost-effective, and without side effects [10,11,12,13]. Many applications have been documented: for example, IVC US guidance gives benefits to patients affected by hypotension [14].

The typical analysis of the IVC by the physician is performed manually, using M-mode visualisation, which allows exploring the vein over time along a single US line. The maximum and minimum IVC diameters (max(D) and min(D), respectively) are measured over a respiration cycle and used to compute the caval index (CI):

(1) $$CI=\frac{max(D)-min(D)}{max(D)}$$

The *CI* provides information about the collapsibility of the investigated blood vessel, reflecting its mechanical characteristics, tissue compliance (both of the vessel and of the surrounding tissues), and transmural pressure [5].

Spontaneous respiratory activity produces an important pulsatile component in the dynamics of IVC. Moreover, heartbeats induce an additional oscillatory dynamics at a higher frequency. By filtering the IVC diameter in different frequency ranges, it is possible to isolate these two important oscillatory components and estimate different collapsibility indexes: the cardiac *CI* (*CCI*) and the respiratory *CI* (*RCI*). As expected, the *CCI* and *RCI* provide information that is, at least in part, uncorrelated [15].

The estimation of the *CCI* and *RCI* requires the IVC diameter to be estimated on all frames, to obtain a time series that can be processed by filtering in order to separate the two oscillatory components. However, segmenting all frames is not feasible by the manual investigation applied in clinics, so that an automated processing of the US videos is required.

Moreover, in the M-mode view usually applied in clinics, the IVC is intersected by a fixed acoustic line, but it moves due to respirophasic cycles. Thus, the clinician’s estimation of IVC diameter is taken on different parts of the vein [16]. This introduces an error, which depends on the longitudinal changes in the shape and collapsibility of the IVC, as well as on its respirophasic movements (e.g., rotations with respect to the acoustic line) [16]. As an alternative to M-mode, brightness mode (B-mode) can be used to visualise a two-dimensional image by interpreting the intensity of the US echoes returning from different tissues. Due to its capacity to present real-time, in-depth structural information on the body’s internal organs and tissues, this modality is frequently utilised in medical imaging. Different IVC segmentation methods have been proposed to track the movements and delineate the vessel walls by processing B-mode US scans [10,16,17,18,19]. Furthermore, these methods reduce the variability associated with the *CI* measurement [15].

In this study, we examined the pros and cons of the current state-of-the-art IVC segmentation techniques in B-mode for both longitudinal and transverse views. Finally, we discuss a number of scenarios in which an automated IVC segmentation could enhance medical diagnostic and prognostic accuracy.

## 2. Anatomy of the IVC

The IVC is the largest retroperitoneal vein in the abdominal cavity. The right and left common iliac veins join to form the IVC, often at the level of the fifth lumbar vertebra. The IVC ascends to the right of the vertebrae and the aorta. The gonads, the suprarenal vein, the left and right renal veins, the hepatic veins, and the lumbar vein are other sources of deoxygenated blood that it collects. The IVC passes through the caval foramen of the diaphragm before entering the right atrium of the heart. It is important to note that the azygos vein, the lumbar veins, and the spinal venous plexuses connect the IVC to the superior vena cava.

The IVC is typically about 22 cm long and 2.5 cm in diameter in healthy adults (with a range of diameter from 1.8 cm to 3.2 cm [20]). The term “mega cava” refers to an IVC with a diameter bigger than 40 mm [21]. A larger diameter may indicate conditions such as congestive heart failure or cirrhosis of the liver. A smaller diameter may be a sign of hypovolaemia or constrictive pericarditis.

The anatomy of the IVC can present some abnormalities in 8.7% of the world’s population [22,23,24]. These variations can occur during its genesis, which corresponds to the period between the 4th and 8th week of gestation, due to the complexity of the generation of vessels [23]. Specifically, the IVC consists of four segments generated from the anastomoses of various vessels: hepatic, suprarenal, renal, and infrarenal. The hepatic segment is derived from the vitelline vein, whilst the suprarenal segment comprises a section of the right subcardinal vein that does not undergo regression. The renal segment is formed via anastomoses of the right suprasubcardinal and postsubcardinal veins, whereas the infrarenal segment originates from the right supracardinal vein. The subcardinal and supracardinal veins gradually replace the postcardinal veins, which persist as the common iliac veins within the pelvis [25,26].

The generation of the IVC is a complex process, which may lead to the creation of anomalies. In individuals with other cardiovascular defects, anomalies of the IVC have been described more frequently [25]. The most-frequent variants are the duplicated IVC and left IVC. Both supracardinal veins remain in a duplicated IVC, a rare variant affecting 0.2–3% of people [27]. The majority of these anatomical variations are asymptomatic, but their identification is fundamental for the correct planning of a complex operation with no side effects [22]. US systems are used to identify these variations; however, other techniques with possible adverse effects, such as computed tomography (CT), which uses ionising radiation, or higher costs, such as magnetic resonance, are preferred because US analysis is considered user-dependent [22].

## 3. Processing US Scans of IVC

**Figure 1:**
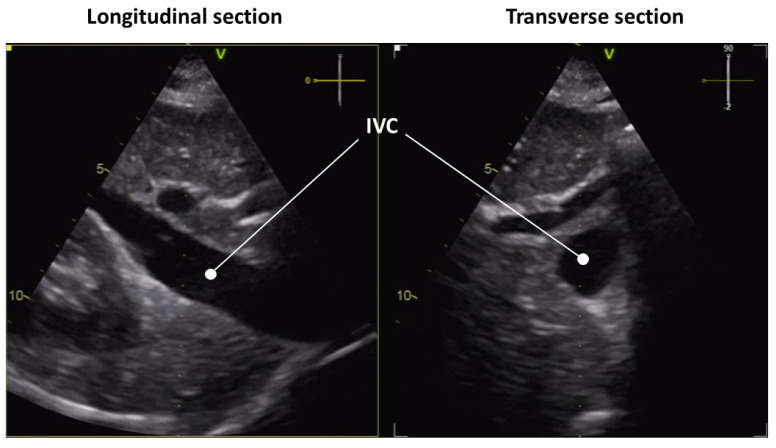
US scan of the IVC in longitudinal (**left**) and transverse (**right**) view.

Various image-processing methods have been applied to US scans of the IVC. Table 1 lists the main methods. The number of algorithms is slightly larger for the analysis of transverse than longitudinal view. This may stem from the fact that it is easier to segment a closed cross-section than an open long-axis portion of the IVC, as the latter requires careful tracking of the region of interest (Figure 1). Furthermore, as shown in Table 1, in recent years, deep learning approaches are gaining more importance, so that further developments are expected in the future in such a direction.

A review of segmentation and identification methods is provided below. The methods are presented grouped according to the view of the IVC they can treat: longitudinal, transverse, or both.

### 3.1. Longitudinal View

#### 3.1.1. KLT Feature Tracker

Kanade–Lucas–Tomasi (KLT) features [33] in association with pyramidal segmentation were used to track the boundary of the IVC over time in longitudinal view [31]. Typical feature-tracking algorithms are computationally expensive because they attempt to match pixel intensity within an area between successive frames. The KLT approach uses spatial intensity information to focus the search for the motion vector that most-accurately captures the feature’s location shift from one frame to the next. Typically, this method is used to track specific image features, such as edges, so that it could fit the problem of IVC edge tracking. However, due to mathematical assumptions, the motion vector must be small, whereas the IVC can show large movements. In order to circumvent this problem, a pyramid segmentation method for image sub-sampling has been used [31]. This way, by analysing the KLT features at multiple resolutions, a large motion vector can also be detected.

To start the analysis, the user must select a pair of points on the edge of the IVC in the first frame of the video to be analysed. The algorithm was able to process a B-mode US video of 10–15 s in 20 s (the algorithm was developed in MATLAB; unfortunately, the hardware used for the analysis was not mentioned). Small discrepancies (<1 mm) were observed when comparing the diameters measured by the physician in M-mode with those calculated by the algorithm. In addition, the raw diameter was low-pass-filtered at a frequency of 0.6 Hz to identify the respiratory component. The cardiac component was obtained as the difference between the raw diameter and the respiratory component.

In summary, the main advantage of this technique with respect to other feature trackers is the small computational cost; however, a single diameter is measured; moreover, to start the analysis, the initialisation of the operator is required.

#### 3.1.2. IVC Tracking and Detection of Intensity Jump

A semi-automatic algorithm, developed in MATLAB, was proposed in [16] to identify the variation of the IVC diameter over time from the longitudinal view. The algorithm tracks the IVC, compensating for its movements and, thus, estimating the diameter, not along a direction fixed in space (as in the case of the clinical approach based on M-mode scans), but integral to the tissues. The software requires the user to provide the following inputs on the first frame:

- Two reference points to be tracked;
- The section along which to estimate the diameter.

To perform the tracking, the algorithm aligns regions (of 128 × 128 px) between consecutive frames in the 2D Fourier domain centred on the reference points and calculates the displacement (three frames are used from the third frame on, to stabilise the estimation). The maximum displacement of the reference points in a successive frame is set to 20 px.

Taking into account the displacement calculated by the reference points, the segmentation line is relocated and the IVC edges are estimated along it. Specifically, a 1D function was obtained by interpolating the intensity values along the segmentation line. After normalisation, an arctangent function was applied to emphasise the rapid changes. The local maximum absolute derivative of the 1D function was identified as the boundary point.

This method was generalised later to explore an entire region of the IVC instead of a single segmentation line [18]. Indeed, the IVC can have a variable collapsibility along its longitudinal course, due to the surrounding structures [34]. Thus, a single segmentation line may not represent the mean collapsibility of the vein and could also be affected by noise, which is reduced by considering an entire portion.

The new algorithm analysing multiple sections of the IVC, to process the first frame of the US video, requires a preliminary interaction with the operator [18], who is asked to:

- Select a rectangular area on the first frame of the US scan around the IVC;
- Establish two landmarks to track tissue movement;
- Identify the first and last segmentation lines that delimit the vein portion of interest and indicate the edges along the first one.

Then, the algorithm generates a number of lines equally spaced between the two selected sections. The IVC boundaries are identified along each of these lines, by selecting points separating regions with low and high intensities (i.e., a darker region and a lighter one are searched, in which the IVC lumen and surrounding tissues are expected, respectively). To reduce the errors in the estimation caused by noise, the location of the boundary points are smoothed, taking into account their neighbours. From the next frame, the following steps are implemented:

1. The reference points are tracked;
2. The segmentation lines are displaced and rotated proportionally to the movement of the reference points;
3. Edges are identified by the algorithm described above, searching for local intensity variations close to the border points found in the previous frame.

This algorithm, developed in MATLAB, was able to analyse a frame of US video in about half a second, when run on an average PC.

By working on an entire IVC segment, a more-consistent and -accurate result can be extracted with respect to investigating a single direction. Moreover, the segmented portion of the IVC was also processed in order to measure diameters in a direction orthogonal to the midline of the vein. The artefacts caused by relative motions between the probe and the IVC and the dependence of the output on the initialisation of the operator were reduced. Furthermore, the outputs were found to be more repeatable, reducing both inter- and intra-operator variability [35].

### 3.2. Transverse View

#### 3.2.1. Active Contour

An active contour, also called a snake, is a curve obtained by minimising an energy functional through a process that forces it to approach a target element in the given image (e.g., an edge) by satisfying internal constraints that impose certain restrictions on its maximum deformation (as if it were a rubber band that exhibits a certain resistance to stretching and bending, finding a certain configuration based on an external force that pulls it towards the target of interest) [36]. Terms relating to the picture gradient, contour smoothness, and contour length are frequently included in the definition of the energy functional. A number of nodes, which are located on the edge curve, have a value for each term of the energy functional. Through the energy-minimisation procedure, each node of the active contour may effectively locate the contour of the object of interest. Because these models can adjust to the shape of the objects, they are very helpful for recognising and tracking their boundaries in pictures, including US images [37]. The segmentation of US pictures can be difficult because of speckle noise, poor contrast, fuzzy or unclear boundaries, and other abnormalities that are frequently present [28]. However, active contours are important techniques for US image segmentation [28,38], due to their capability to take into account a priori knowledge regarding the shape and smoothness of the object borders (as mentioned above).

A specific method was developed to segment the IVC in transverse view using a snake [10]. The energy functional was defined as follows, based on binarised frames:

(2) $$E_{i}=\alpha_{i}E_{cont,i}+\beta_{i}E_{curv,i}+\gamma_{i}E_{img,i}$$

$E_{cont,i}$ represents the energy of continuity of the *i*th point that constitutes the border. The aim of this component is to force a uniform distribution of the points of the snake. The second component is the energy of curvedness $E_{curv,i}$; it penalises the curvature of the estimated contour. The last component, $E_{img,i}$, is the energy of sharpness; it is the edge gradation strength, which depends on the sharpness of the image.

A greedy algorithm was used to minimise the energy functional after a double-dilatation and erosion on the US image. As a final step, to obtain a smoother border, the estimated snake was approximated by an ellipse. The operator needs to select a point inside the IVC to start a segmentation. Over the eight subjects tested in [10], the snake was able to segment only four. However, the algorithm performed the analysis in real-time.

In [28], a polar active contour algorithm was proposed, with a particular energy functional to improve the performance of the IVC segmentation in the transverse view. Assuming a convex shape of the IVC in the transverse view, its border could be described in the polar coordinates. The following energy functional is minimised to identify the IVC edge:

(3) $$E=E_{M3}+E_{curv}$$

$E_{M3}$ is proportional to the third centralised moment of the object:

(4) $$E_{M3}=-\alpha \frac{\int_{C}{\left(I-\bar{I}\right)}^{3}dA}{\int_{C}dA}$$

where α is a positive weight, *I* is the intensity image, and $\bar{I}$ is its mean inside the circle *C*. This term is computed over circles with different radii and finds the minimum at the border of the IVC. The second term in the energy defined in Equation (3), i.e., $E_{curv}$, measures the curvature of the estimated contour.

The implemented software was semi-automated, as, to start the analysis, it required that the user indicated the central point of the IVC. The algorithm was tested against manual delineation and four other segmentation algorithms. On a tiny dataset composed of two videos, the proposed algorithm outperformed the others and was the only one to approximate the variations over time of the area of the IVC observed in the manually segmented frames.

In general, there are three limitations of active contour approaches: it is difficult to obtain a real-time implementation, and it requires the tuning of the parameters for convergence and a careful initialisation [37]. Moreover, the snake needs some initialisation to start a segmentation, and local minima can stop the identification of the IVC border.

#### 3.2.2. Estimation of Jumps of Intensity along Lines

An alternative segmentation algorithm for transverse IVC segmentation, implemented in MATLAB, was proposed in [19]. The operator selects the central point in the IVC lumen, and 20 radially distributed segmentation lines are automatically generated to identify the vein boundary. The intensity values along each line are approximated by a step function, assuming that it is darker inside the IVC and brighter outside. When considering a new frame, the border along each line is searched in a range of 20 px centred around the point detected in the previous frame. After border estimation, in order to smooth it, a low-pass filter is applied to its *x* and *y* coordinates.

Given the border of the IVC cross-section, the equivalent diameter was computed as

(5) $$D=2\sqrt{\frac{Area}{\pi }}$$

This allowed obtain a more-stable estimation of the diameter with respect of using a measurement taken along a single direction. The cardiac and respiratory components of the IVC collapsibility were also separated (using a Butterworth filter of order four and cut-off frequency of 0.4Hz) and investigated [19].

#### 3.2.3. Shape-Based Algorithms

Clinical studies have demonstrated that a patient’s volume status and the effect of extra intravenous fluids may be roughly estimated from fluctuations in the IVC’s anterior–posterior (AP) diameter. Such an AP direction usually allows observing the largest mean diameter of the IVC, which typically has an elliptical cross-section.

Some algorithms were developed assuming a specific shape of the IVC cross-section. In [8,29], the following error functional was minimised to find the IVC border:

(6) E=α(u−v)(2I−u−v)

where α is a positive parameter, *I* is the local intensity, and *u* and *v* represent the mean intensity inside and outside the IVC, respectively. A circular model was used, as it has the advantage of reducing the number of variables to be estimated, that is only the radius of the circle [8]. The algorithm performed well, with the AP diameter estimation quite similar to the ones measured manually by an expert.

In [29], the same function (6) was used, but assuming either a rectangular or an elliptical shape of the IVC. Finally, a comparison between the three algorithms, i.e., those assuming either a circular, rectangular, or elliptical shape, was discussed. The circular algorithm showed some bias in the diameter estimation. Interestingly, the best performances were obtained by assuming the rectangular shape, both in diameter estimation and in computational cost (in fact, fewer iterations were needed to reach convergence). Moreover, the algorithm assuming a circular IVC was able to obtain a better diameter estimation than when assuming an elliptical shape.

All methods are semi-automated, with the user needing to select a point inside the IVC to start the analysis.

### 3.3. General Methods

#### 3.3.1. Block-Matching Algorithm

Speckle noise, caused by the backscattering of US waves generated by the transducer, is considered an intrinsic feature of corrupted US videos and a form of multiplicative noise [39]. The granular appearance of images that contain speckle noise reduces the contrast, making them difficult to analyse for both clinicians and post-processing algorithms. Several algorithms have been developed to reduce speckle noise due to its negative impact on image analysis [39,40].

However, the speckle patterns in each image region are unique and typically stable in consecutive frames [17]. This makes them a useful feature to compare in different frames in order to solve a tracking problem. In fact, it has found many application in echocardiography, to measure the strain of the myocardium [41].

A block-matching algorithm was proposed also to track speckle noise in the longitudinal view of US videos of the IVC [17]. A number of blocks were placed around the edge of the IVC. The zero-mean normalised cross-correlation of pairs of frames was maximised with respect to the displacement to track the upper and lower margins of the IVC. The interpolation of points was performed using a cubic curve. The algorithm was able to successfully segment 142 US videos on an initial dataset consisting of 190 patients.

A similar approach was proposed in [10] for edge detection of the IVC in transverse view. In the first frame (*T*), the operator was instructed to mark seven points on the border of the vein. Subsequently, the captured frames (indicated with *I*) were analysed, whereby the new location of each border point was calculated by minimising the squared differences:

(7) $$R_{ssd}=\sum_{j=0}^{N-1}\sum_{i=0}^{M-1}{\left(T\left(i,j\right)-I\left(i,j\right)\right)}^{2}$$

The search area was configured to 40 × 40 px. Once the tracked points were identified in the next frame, an ellipse was fit to estimate the border of the IVC. The algorithm successfully segmented seven out of the eight US videos considered in [10], demonstrating its superiority over the active contour approach described in the same paper. However, compared to the active contour approach, which requires only one point, the main disadvantage of this method is that the operator has to select seven points, making the algorithm more dependent on the inputs from the user. In addition, due to the high computational cost, the analysis can only be performed offline.

#### 3.3.2. Deep Learning

In recent years, thanks to regularisation techniques such as drop-out [42], a subset of machine learning, which has the peculiarity of using many layers of neurons, known as deep learning, has become a thriving technology for data processing [43]. Medical imaging in general is one of the fields where extreme improvement is achieved by such models [44,45,46]. Deep learning is effectively applied to point-of-care US (POCUS) imaging due to its wide range of applications and diverse user base with different levels of training [47].

Convolutional neural networks (CNNs) have been used extensively in a wide range of applications, including image recognition [48,49], brain–computer interfaces [50,51], natural language processing [52,53], and medical image analysis [54,55,56,57].

Long short-term memory (LSTM) is a special type of recurrent neural network that allows considering also the information related to time, in particular the long-term dependencies [58]. For the analysis of US scans of veins, time dynamics is a really important feature: including information on IVC movement (instead of using a static image) improves its identification [30].

An important achievement in the treatment of critically ill patients is to improve the cardiac output (*CO*), that is the product of the heart rate (*HR*) and stroke volume (*SV*):

(8) CO=HR·SV

A deep learning model was used to classify subjects as responders or not responders using US video of the IVC acquired in the cranio-caudal plane [59]. A patient was classified as a responder if a 10% increase in the cardiac index was detected by non-invasive *CO* monitoring after administration of 500 mL of normal saline [59]. The deep learning model consisted of:

- A CNN, specifically a VGG16 [60], due to the good classification performance in US image analysis [32];
- A bidirectional LSTM network.

It was designed to output only the classification between the two classes of interest (without making a vein segmentation). Good performance was achieved, but further studies are needed to expand the dataset.

For expert clinicians, a binary output of interest (e.g., responders or not responders, in the above-mentioned study) may increase the speed of the decision process. However, for novices, vein segmentation can be useful information for learning to identify the IVC and understanding its dynamics in different conditions.

IVC detection on each frame of a US video was explored in [30]: a deep learning model was used to identify the view (either cranio-caudal or medio-lateral) and to detect the IVC. The model offered excellent detection performance. Moreover, real-time processing could be guaranteed for a US video. However, the algorithm simply identifies for each frame the position of the IVC within an ROI; thus, it does not provide the border segmentation, nor any indication of collapsibility. However, this method could be useful to bypass the preliminary steps needed to start the segmentation of the vessel by one of the methods discussed above, with the aim of developing a fully automatic method for IVC segmentation.

Guiding devices and catheters through the vasculature during transcatheter cardiac interventions is another area where IVC segmentation is gaining importance.

A deep learning model (U-net [61]) was used to create a non-invasive (US-based) alternative to fluoroscopy for catheter guidance [62]. The model was tested on animals and reached a segmentation accuracy of 90%. As prospects, transcatheter cardiac procedures could be performed with this fluoro-free image-guided system, which can guide instruments and catheters through the vasculature, using this US-based segmentation in conjunction with electromagnetic tracking technology.

Finally, there could be approaches that are currently being applied to the segmentation of arteries, but which could also be applied to veins. For example, IVUS-net [63] is a fully convolutional network that was able to segment US images, identifying the lumen and media-adventitia border of the arteries. It is based on U-net and SegNet [64] and is composed of an encoder, which generates the feature maps with a lower resolution compared to the original image, and a decoder, which restores the original image size starting from the feature maps. The segmentation performance is interesting and fast (6.6 frames per s).

## 4. Clinical Applications of IVC Diameter Estimation

**Figure 2:**
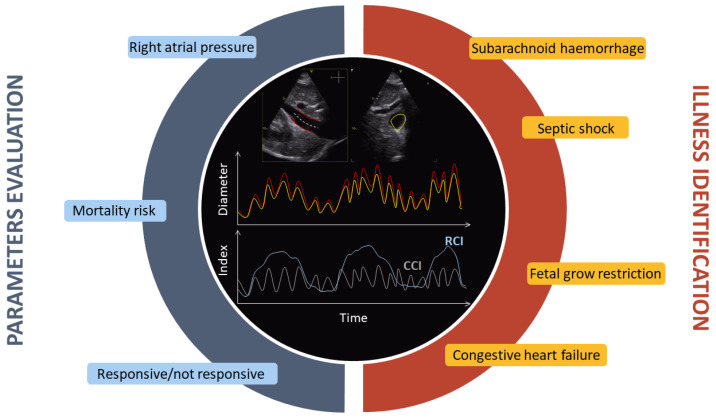
In the top and centre, there are two views of the IVC shown in red and yellow: longitudinal (**left**) and transverse (**right**). Below are the corresponding estimated diameters over time. At the bottom, the *CCI* and *RCI* parameters are shown as measurements of collapsibility. The main applications are indicated all around, divided into two sections: parameter evaluation and illness identification.

This section discusses the applications of US scans of the IVC. Figure 2 shows that both the IVC diameter and the collapsibility index extracted from it can be used for the estimation of particular parameters or to identify the severity of a pathological condition. These applications are explained in the following.

### 4.1. Foetal Grow Restriction

Foetal growth restriction (FGR) is a medical condition where the developing foetus does not grow at the expected rate during pregnancy. FGR can result in complications during pregnancy and childbirth, and it can increase the baby’s risk of health problems, both in the short and long term. In severe cases, FGR can lead to stillbirth or neonatal death [65]. FGR is a common problem, affecting 10–15% of pregnant women [66]. The most-common technique for identifying FGR is Doppler US. However, its poor ability to distinguish between pathological and constitutional conditions has led clinicians to identify new techniques and parameters [66,67]. One study involving 176 pregnant women found that, with an increase in the severity of FGR, the diameter of the IVC and aorta decreased [66]. This indicates an interesting application of US scans, which can determine the sizes of those vessels.

### 4.2. Right Atrial Pressure

The pressure of the blood in the right atrium of the heart is referred to as the right atrial pressure (RAP). This pressure is linked to both the volume of blood that returns to the heart and the heart’s capacity to pump the blood into the arterial system. The RAP can often be nearly identical to the central venous pressure (CVP). However, the two terms are not equivalent, since there can occasionally be a difference in pressure between blood in the vena cava and in the right atrium.

The RAP is conventionally measured after exhaling, using invasive approaches (e.g., right atrium catheterisation) and ranges between 1 and 7 mmHg in healthy people [68,69], but it can be higher than 20 mmHg in pathological conditions [70]. Due to its invasiveness, this measurement is performed only in a subset of patients under particular conditions. New non-invasive techniques are currently being developed to standardise the evaluation of the RAP and make it a routine measurement [5].

US imaging of the IVC is a widespread non-invasive technique used to measure the RAP by analysing its diameter and changes over the respiratory cycle, as stated by several sources [5,13,70,71]. However, some studies have suggested that there is only a modest correlation between the RAP measured invasively and using echocardiography, along with issues surrounding the reproducibility of IVC assessment by US [72,73]. This modest correlation may be influenced by several factors. For instance, operator dependence in the US scan and measurement of the IVC diameter could be some of them. An automated segmentation algorithm for both transverse and longitudinal sections can offer a reliable assessment of the size and collapsibility index of the IVC. Preliminary studies suggest that integrating information from different indexes extracted by automated processing of IVC US videos can improve the estimation [5,13,70]. In addition, it has been shown that multi-parameter prediction models utilising IVC diameter, the cardiac index, the corrected flow time index, and the right ventricular ejection fraction lead to an improvement in the estimation of the RAP [70].

Regrettably, conflicting results have been reported over the years regarding the assessment of the RAP through the IVC. Although some studies have confirmed that the estimated RAP obtained from M-mode US videos of the longitudinal view of the IVC is positively associated with end-respiratory IVC diameter, the correlation is very weak [74].

### 4.3. Fluid Administration

Fluid management is an important task in intensive care units. Determining the appropriate amount of fluid for each patient is crucial [7,75]. Furthermore, proper fluid management prevents problems resulting from excessive fluid volume [76,77] including acute pulmonary oedema [78]. Nonetheless, only 50% of patients treated with fluid administration exhibit positive outcomes [79,80].

Fluid resuscitation, which involves administering fluids and electrolytes, is mainly intended to increase *CO* and preserve organ perfusion and substrate supply (including oxygen). If oral intake is not feasible, clinicians can compensate for fluid loss through intravenous administration. According to [81], a patient is considered a responder when the administration of a 500 mL fluid bolus results in a 15% increase in *CO* within 10–15 min. Regrettably, only 50% of patients respond to fluid administration with an increase in *SV* [82]. Patients who do not respond to the fluid bolus are more likely to develop fluid overload [83].

The most-precise way to guide fluid administration decisions is by using dynamic methods that calculate the change in *CO* resulting from a fluid bolus. Unfortunately, the application of dynamic measures is still limited due to the required technical expertise, expensive equipment, and applicability to only a small sample of patients [84].

POCUS has attracted attention as a potential tool to assist clinicians in prescribing fluid therapy due to its rapid, repeatable, and non-invasive nature. US image analysis can measure vessel diameter and collapsibility during passive leg raising (PLR), providing significant information to predict fluid responsiveness [82]. Studies show that the cardiac component of IVC collapsibility is less affected by the spontaneous variability of respiratory pattern and is, therefore, a more-robust indicator [17]. A new, more-stable index for studying vessel collapsibility has been proposed as the averaged *CCI* over different respiratory cycles, known as the *aCCI* [15].

Clinicians can make use of these closely related indicators of dynamic assessments of fluid responsiveness to guide fluid resuscitation in critically ill patients [85]. Recent studies have demonstrated that venous collapsibility is directly correlated with the diameter of the IVC and may be inversely associated with the CVP, where a change of 1 mmHg in the CVP corresponds to a 3.3% change in IVC collapsibility [86].

The administration of fluids is crucial in the event of septic shock. Sepsis is characterised by organ dysfunction due to a dysregulated host response to infection [87]. In sepsis, the body overreacts to pathogens (viruses, fungi, bacteria) by releasing excessive amounts of inflammatory molecules. The release of inflammatory mediators causes a decrease in the venous return and dilatation of arterial and venous vessels, ultimately leading to hypotension and distributive shock. Arteries, veins, and capillaries undergo dilation, and there are significant changes in endothelial permeability, leading to intravascular fluid leakage [88]. Impaired tissue perfusion causes organ dysfunction. Fluid resuscitation is used to enhance tissue perfusion and prevent organ dysfunction [88].

POCUS may aid clinical judgement in septic patients, particularly regarding fluid management [84,89]. Several scientific articles have confirmed the utility of parameters extracted from the IVC in predicting fluid responsiveness in mechanically ventilated patients with sepsis [90,91].

### 4.4. Volume Conditions

Evaluating the degree of vascular filling, known as intravascular volume status, is crucial for the assessment of the cardiovascular system. In fact, it is closely connected to the cardiac preload, which is the stretching of the cardiac tissue in a relaxed state and directly affects both *CO* and arterial blood pressure.

Evaluating the intravascular volume status in critically ill patients is of utmost importance in determining and maintaining the optimal balance in fluid therapy. Although fluid supplementation can enhance cardiac efficiency, it may also increase the risk of complications and mortality [92].

Several pathological conditions result in a reduction of the systemic venous return, leading to a hypovolaemic state in the patient. A reduction in the systemic venous return and, consequently, in the *CO* can be observed in these patients [93].

Additionally, the primary cause of death following an injury is massive haemorrhage [94]. Subarachnoid haemorrhage (SAH) is a severe cerebrovascular condition, which occurs when an intracranial aneurysm ruptures, leading to a haemorrhage into the subarachnoid space. An abnormally low intravascular volume state has been observed in most SAH patients, and the degree of hypovolaemia is correlated with the clinical grade [95].

Identifying hypovolaemic status is a complex task, although prompt recognition (class-I hypovolaemic shock, loss of 450–500 mL) is necessary to achieve a favourable treatment outcome. Laboratory parameters, such as metabolic acidosis, high urea, haemoconcentration or hypotension, tachycardia, or signs of tissue hypoperfusion are insufficient in detecting this specific status [93]. A new parameter, based on the diameters of the IVC and aorta and obtainable through US imaging, was proposed to identify class-I hypovolaemic shock [93]. The parameter was tested on 52 healthy blood donors and achieved promising results in identifying blood volume reductions.

Evaluating non-invasively the volume status (especially hypovolaemic status) is crucial as it facilitates examining patients in emergency situations or during follow-up appointments. An innovative method was developed to identify the hydration state using binary tree models trained on the collapsibility of the IVC, extracted by segmenting B-mode images over time [92]. Indeed, a correlation was found between the intravascular fluid volume and the collapsibility of the IVC [96]. A significant association was found between the initial diameter of the IVC and the requirement for massive transfusion in patients experiencing blunt trauma [97]. This evidence suggests that integrating the evaluation of IVC diameter into clinical practice can potentially enhance patient outcomes by enabling early recognition and treatment of patients at higher risk of requiring significant blood transfusions [97]. It is worth noting that imaging was performed using CT in [97]. However, the diameter evaluation of the IVC could also be achieved through non-invasive US scans, which sample at a high frame rate, allowing exploring also dynamical features such as collapsibility indexes.

### 4.5. Congestion and Heart Failure

Congestive heart failure (HF) is characterised by fluid accumulation in the intravascular compartment and interstitial space. It is a consequence of increased cardiac filling pressures due to maladaptive retention of sodium and water by the kidneys. This congestion is the major cause of hospitalisation in patients with acute decompensated HF [69,98]. According to the European Society of Cardiology Heart Failure Long-Term (ESC-HF-LT) registry, which includes data from 16,012 patients, clinical signs or symptoms of congestion, such as dyspnoea, rales, and peripheral oedema, were present in 83% of all patients hospitalised for acute HF [99]. If a patient with congestion is not treated promptly or is discharged from the hospital too early, this can further increase the risk of death or rehospitalisation [69].

The primary method of treating congestion is to reduce the amount of blood in the circulation with loop diuretics [69,100]. Sodium excretion and urine output are increased because these drugs inhibit sodium reabsorption. However, the accurate detection and quantification of congestion is difficult, but essential for the correct management of the patient [101,102]. Measurement of the RAP and pulmonary capillary wedge pressure (PCWP) are two methods commonly used to assess congestion [69,103]. High RAP values predict poor outcome in patients with HF [9]. These measurements, which are intrusive, cannot be used in all patients or for subsequent medical management. However, as reported in the previous section, the RAP could be estimated from IVC analysis.

Furthermore, IVC dilatation after acute decompensated HF (ADHF) can be used as a marker of high mortality risk [5,101,102,104,105]. Indeed, in patients hospitalised for ADHF, an IVC diameter greater than 21 mm is a measure of all-cause mortality in patients with renal failure [104]. According to [101], increased IVC diameter or the number of B-lines (i.e., in lung US, perpendicular signals emanating from the pleura, often indicating the presence of extravascular lung water) can be used to identify individuals with chronic HF who are more likely to have worse outcomes and have higher plasma concentrations of NT-proBNP, a biomarker used to diagnose HF released from the ventricles in response to increased wall stress.

## 5. Future Perspectives

Currently, US recording systems are mainly used by professionals, particularly medical doctors. This is mainly due to the large size of most devices and the need to use cables to connect the probe to an acquisition, processing, and display system (usually a PC). Furthermore, they need great experience to be correctly used: for example, there is a need to manually position the probe, point it at anatomical details, which require experience to be recognised, and handle it by accommodating the patient’s movements. This makes US scans operator-dependent and requires specialised medical professionals for analysis and interpretation [106].

However, novel devices for US scans have been developed recently, which are small and allow for wireless connection. There are also devices with the capability to record for up to 12 h and transmit them to either a smartphone or computer, for processing and storage [106]. This opens the opportunity for monitoring the patient continuously, for a long time range. The wearable ultrasonic-system-on-patch (USoP), in combination with appropriate software, may represent a pivotal advancement for personalised medicine. Furthermore, patients can be monitored without the need for the constant presence of an expert, whether in a hospital or at home. Processing data to extract essential information (e.g., the edge of a vessel of interest) could be useful to save resources (e.g., reduce power consumption or memory storage). In addition, potentially dangerous conditions for the patient (e.g., a high RAP) could be identified in real-time and ensure intervention at the right time, which could be decisive in many situations.

## 6. Conclusions

This article provides a comprehensive analysis of various methodologies for IVC segmentation or identification from B-mode US scans, encompassing both longitudinal and transverse views. A detailed examination of the merits and drawbacks of each approach is presented. The significance of IVC studies was underscored through an exploration of its diverse applications in the medical field, where such data can potentially catalyse significant advancements over conventional methodologies.

In conclusion, the advent of algorithms that autonomously identify and segment the US scans of the IVC can effectively mitigate the primary challenge associated with the analysis of images in US: operator dependence. Furthermore, by standardising the parameters and measurements, the diagnostic and prognostic efficacy of clinicians can be improved, facilitated by the availability of more-reliable and -consistent data. 

## Figures and Tables

**Table 1:** Segmentation and identification algorithms for the IVC grouped according to the view that they can treat, i.e., transverse and longitudinal views. The authors, publication dates, and main methods used to obtain the segmentation are reported.

IVC View	Article	Publication Date	Method
Transverse	[10]	2013	Snake
	[10]	2013	Template matching
	[28]	2017	Snake
	[8]	2018	Shape-based algorithm
	[29]	2019	Shape-based algorithm
	[19]	2020	Jump of intensity
	[30]	2023	YOLO
Longitudinal	[16]	2015	Tracking and jump along a single line
	[17]	2015	Block matching
	[31]	2018	KLT features
	[18]	2019	Tracking and jumps on multiple lines
	[32]	2020	CNN and LSTM
	[30]	2023	YOLO

## Data Availability

Not applicable.

## Abbreviations

The following abbreviations are used in this manuscript:

ADHF	Acute decompensated heart failure
AP	Anterior–posterior
CCI	Cardiac caval index
CNN	Convolutional neural network
CO	Cardiac output
CT	Computed tomography
CVP	Central venous pressure
ESC-HF-LT	European Society of Cardiology Heart Failure Long-Term
FGR	Foetal grow restriction
HR	Heart rate
IVC	Inferior vena cava
KLT	Kanade–Lucas–Tomasi
LSTM	Long short-term memory
PCWP	Pulmonary capillary wedge pressure
PLR	Passive leg raise
POCUS	Point-of-care ultrasonography
RAP	Right atrial pressure
RCI	Respiratory caval index
SAH	Subarachnoid haemorrhage
SV	Stroke volume
ROI	Region of interest
US	Ultrasound
YOLO	You only look once
USoP	Ultrasonic-system-on-patch
